# Hospitals and the Spirit Tax: The Government Amendment to the Finance Bill

**Published:** 1915-06-26

**Authors:** 


### HOSPITALS AND THE SPIRIT TAX.

### The Government Amendment to the Finance Bill.

As briefly indicated in last week's issue of The Hospital (page 248), the idea of duty-free alcohol for hospitals appears to be on the point of realisation, a Government amendment to the Finance Bill which provides for this having been tabled. It was expected that the new clause which embodies this proposal would have been considered in the House of Commons on the Committee stage of the Bill on Thursday last week, but the discussion on the new clauses was postponed, and at the time of writing no further progress had been made. There is, however, every reason to hope that before the Finance Bill passes into law it will include the reasonable concession as to the use of duty-free spirits in hospitals which is contained in the proposed new provision. The text of this clause was printed in The Hospital last week, but as the matter is one of great importance it will be useful to repeat it. It is as follows:

### Text of the New Clause.

(1) Section 8 of the Finance Act, 1902 (which gives power to authorise the use of spirits without payment of duty in arts and manufactures), shall apply to the preparation in a public hospital of tinctures and other articles to be used for medical purposes in the hospital as it applies to arts or manufactures in which the use of spirits is required.

(2) If the treasurer or other responsible officer of a public hospital shows, to the satisfaction of the Commissioners of Customs and Excise, that any tinctures or other articles which contain spirit, or in the preparation or manufacture of which spirits were used, have within the preceding six months been used for medical purposes in the hospital, he shall be entitled to obtain from the Commissioners repayment of any duty paid in respect of the spirits contained in or used in the preparation or manufacture of the said tinctures or other articles.

If any person, for the purpose of obtaining any repayment under the foregoing provision, knowingly makes any false statement or false representation, he shall be liable, on summary conviction, to imprisonment, with or without hard labour, for a term not exceeding six months.

(3) For the purpose of the foregoing provision the expression "public hospital" means a hospital supported by any public authority, or wholly or partly out of any public or charitable funds, or by voluntary subscriptions.

### A Possible Criticism.

There seems to be no solid reason why this clause should not pass into law in its present form. But some opposition may be anticipated, not to the principle of the proposal, but to the paragraph defining the expression "public hospital." It will probably be contended that the definition is too comprehensive and provides a loophole for abuse. Hospitals "supported by any public authority," or "wholly supported out of any public or charitable funds," or "wholly supported by voluntary subscriptions" would no doubt pass the critics; but the inclusion of hospitals "partly" supported by public or charitable or voluntary subscriptions may give rise to criticism on the grounds that the broader definition opens up a way through which bogus institutions could obtain concessions which are only intended to be available to *bona-fide* hospitals and infirmaries. If the danger were a real one there would be good reason to amend this definition, but it can safely be left to the Commissioners of Customs and Excise to discriminate between the institutions that are contemplated by the clause and those that are not. Moreover, the heavy penalty entailed in making a false statement or false representation should be sufficient to deter dishonourable people from jeopardising a concession that is intended to confer benefits upon genuine public hospitals.

### A Simple Procedure.

The whole subject of hospitals and the spirit tax was discussed in The Hospital of May 8, 1915, and various means were suggested, by the adoption of which the Chancellor of the Exchequer could relieve hospitals of a heavy burden. The provisions of the proposed new clause reflect these suggestions in that the clause provides both for the use of rectified spirit, duty free, "in the preparation in a public hospital of tinctures and other articles," and for the repayment of the duty paid on spirits used in the preparation of tinctures and other spirituous medicinal preparations that may have been bought from outside sources. That is to say, whether the authorities of a hospital buy the spirit for making tinctures in their own laboratory, or whether they buy their tinctures ready-made, the spirit will be free of duty; in the first case, no duty will be paid, and, in the second, the amount of the duty will be refunded. The whole procedure is very simple and very secure; the wonder is that the concession has never been granted before. It is probable that it would not have been conceded even now had it not been for the fact that the heaviness of the burden of the spirit duties on hospitals was brought to the notice of the Chancellor of the Exchequer very forcibly when he proposed some short time ago to double the tax on spirit. It was shown to him that doubling this tax meant doubling the duty on over two hundred medicinal preparations prescribed in hospitals, and adding in some cases as much as three hundred pounds a year to the expenditure of a hospital without the remotest possibility of any advantage to the State. The Chancellor declared his intention of preventing this increase, and intimated that even if the extra duty on potable spirits was maintained there should be no increase in the duty on medical spirits. But if it is possible to differentiate between potable spirits and medical spirits to the extent of relieving the latter from half the tax it should not be difficult to carry the discrimination still further and relieve medical spirits used in hospitals from the whole of the tax. This, in fact, is what is purported by the proposed new clause. In a very large measure the thanks of hospital authorities are due to Mr. Bridgeman, the member for Coventry, who as a private member persistently urged the Chancellor to free hospitals from the unnecessary burden. By the decree of fortune Mr. Bridgeman is now a Junior Lord of the Treasury, and the clause which he intended to move from the Opposition benches has been adopted by the Chancellor of the Exchequer. It is sincerely to be hoped that no further political changes will arise to prevent the prompt enactment of a most beneficent provision.

